# Potential of Traditional Knowledge of Plants in the Management of Arthropods in Livestock Industry with Focus on (Acari) Ticks

**DOI:** 10.1155/2017/8647919

**Published:** 2017-07-17

**Authors:** Wycliffe Wanzala

**Affiliations:** ^1^Department of Biological Sciences, School of Science and Information Sciences, Maasai Mara University, P.O. Box 861-20500, Narok, Kenya; ^2^Behavioural and Chemical Ecology Department, International Centre of Insect Physiology and Ecology, African Insect Science for Food and Health, P.O. Box 30772-00100-GPO, Nairobi, Kenya

## Abstract

Antitick plants and related ethnoknowledge/ethnopractices with potential for integrated tick control and management strategies to improve livestock production are reviewed. About 231 plants reviewed showed a variety of bioactive properties, namely, being toxic, repellent, antifeedant, and antiovipositant and ability to immobilize target tick species. These ethnobotanical substances are potentially useful in developing sustainable, efficient, and effective antitick agents suitable for rural livestock farmers. Majority of these plants are holistic in action, economically affordable, user friendly, easily adaptable and accessible, and environmentally friendly and help develop community-driven tick control interventions well suited to local conditions and specific to different livestock communities. Such a multipurpose intervention best fits the recent ascendancy of individual livestock owners as the key players in tick control programmes, particularly following the withdrawal of subsidies accorded to tick control programmes by most African government agencies since mid-1980s. However, scientific validation of antitick ethnobotanicals on their efficacy and formulation of packages easily handled by local communities is necessary to achieve a significantly increased use of such remedies. It is envisaged that the results of validation may lead to the discovery of effective and affordable antitick products. The effectiveness of these “best bets” ethnopractices can be greatest, if they are appropriately blended with conventional technologies.

## 1. Introduction

Animals worldwide are externally infested by a number of parasitic insect and acarine species, collectively called ectoparasites. Ectoparasite, a word originating from the Greek words,* ektos* meaning outside and* parasitos* meaning parasite, refers to an organism that lives on the exterior of its host and to the detriment of that host. These ectoparasites include lice, mites, fleas, blowflies, blackflies, mosquitoes and ticks. They afflict humans and livestock alike, causing major socioeconomic losses and suffering of human life and livestock industry, predominantly as a result of transmission of a wide variety of pathogens (viruses, rickettsiae, spirochetes and bacteria, fungi, protozoa, filarial worms, and nematodes), some causing deadly dangerous zoonotic diseases [1–4]. In addition, they cause skin diseases, annoyance, uneasiness, itching, wounds (source of secondary infections), myiasis, hide/skin damage, reduction of meat, milk, blood, and wool production, and low income from the sales of farm animals and their products [5].

Of these blood-feeding ectoparasites, ticks are the most important arthropod disease vectors, surpassing all other haematophagous arthropods in number and variety of diseases they transmit to animals and humans [6]. By virtue of their protracted feeding period, ticks represent an extreme example of evasion of their host's haemostatic defenses and immune response, thus becoming better placed pathogen transmitters than any other arthropods known [7–12]. The lack of digestive enzymes in the tick gut favours the survival of ingested microorganisms and may explain why ticks transmit a greater variety of pathogens than any other haematophagous arthropods [13]. Notably, a wide range of tick-borne bacterial diseases (rickettsioses, ehrlichioses, Lyme disease, relapsing fever borrelioses, tularemia, and Q fever) and Omsk hemorrhagic fever, louping-ill disease, tick-borne encephalitis, West Nile fever, and Crimean-Congo hemorrhagic fever are increasingly emerging diseases of human concern [14, 15].

Of all ectoparasites infesting livestock, ticks cause the greatest economic losses in livestock production systems at a global level [6, 13, 16]. However, in Africa (particularly in East Africa), tsetse flies, which infest only 40% of the continent [17], surpass ticks, which are found on the entire continent (30 M sq km) in terms of socioeconomic losses incurred in livestock industry. Livestock ticks transmit a variety of aetiologic organisms (bacteria, protozoa, rickettsiae, and viruses) and the causative agents of a number of debilitating livestock diseases (theileriosis, heartwater, Nairobi sheep disease, streptothricosis, babesiosis/piroplasmosis, and anaplasmosis). These diseases, together with the abundance of the individual tick species, are widely distributed globally in tropical and subtropical regions [18]. The most economically important ixodid ticks infesting livestock in these regions belong to the genera:* Amblyomma, Haemaphysalis, Rhipicephalus, Boophilus, Hyalomma, Dermacentor*, and* Ixodes*.

**Figure 1:**
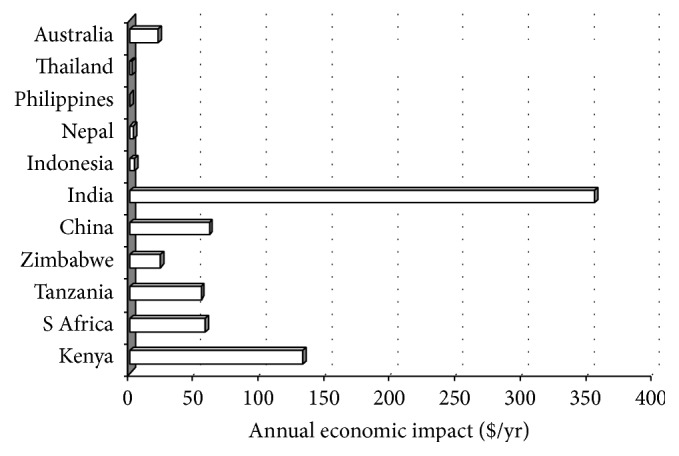
Annual economic impact of tick and tick-borne diseases (US$m) as outlined by McLeod and Kristjanson [19].

**Figure 2:**
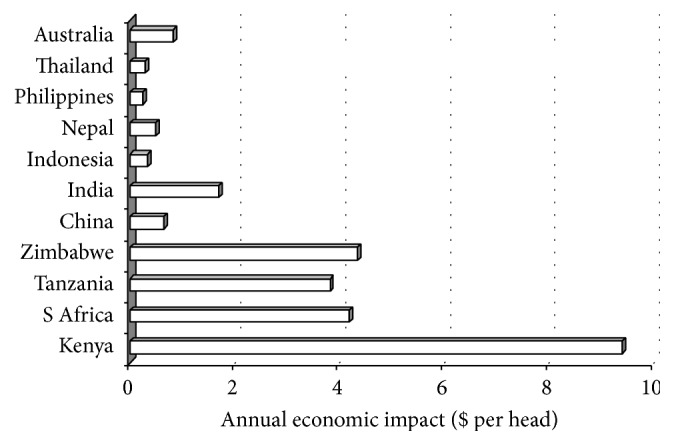
Annual economic impact of tick-borne diseases on a per head basis as outlined by McLeod and Kristjanson [19].

The impact of ticks and tick-borne diseases (T&TBDs) continue to be felt in rural Africa, Asia, and some parts of Americas with untold suffering and losses in livestock and livestock-dependent livelihoods [19]. Estimation of economic impacts of T&TBDs is, however, confounded by lack of accurate estimates of disease prevalence, the heterogeneous nature of cattle production, and complexity associated with the estimation of direct and indirect disease-related production losses [19–22]. However, some annual costs due to T&TBDs control and management in selected countries of Africa and Asia and also in Australia are shown in Figure 1. The economic losses were highest in India ($US 355 million) and lowest in the Philippines. The annual economic costs of T&TBDs per head are shown in Figure 2. They were lower in the Asia-Australia region than in Africa due to the fact that high intensity tick control and management methods are employed in African countries where a highly pathogenic tick-borne disease (East Coast fever caused by* Theileria parva parva*) is endemic [23], causing the highest cattle morbidity and mortality [24, 25].

Despite progress in scientific research and development, T&TBDs' control worldwide has continued to rely heavily on synthetic chemical acaricides. Overdependence on these acaricides diverted attention from exploring and developing sustainable alternative method(s) including traditional methods of tick control and management. Unfortunately, synthetic chemical acaricides have long become unsustainable to use in T&TBDs' control and management interventions [23, 26–29]. Such compounds have suffered from a number of drawbacks, including acaricide resistance in ticks, their rocketing costs, pollution of the environment and food products (meat, blood, and milk) with toxic residues, deleterious effects on nontarget organisms, creation of an enzootically unstable disease situation, and the uncertainty of new acaricidal molecules being produced in the near future due to prohibitive costs of investment in research and development by the manufacturing firms [30]. Additionally, the efficacy of some acaricides/ectoparasiticides against some ectoparasites became questionable [31]. Societal and scientific concerns regarding exclusive dependency upon synthetic chemicals have emphasized the need for the development and introduction of alternatives to acaricides that are consistent with the principles of sustainable agriculture [32]. However, the alternative tick control interventions that exist, namely, use of ethnobotanicals including antitick pastures, biological mechanisms (parasitoids, predators, microbial agents, and nematodes), establishing endemic stability for tick diseases, manipulation of hybrid sterility between closely related tick species, hand deticking, habitat modification, pheromone- and host odor-mediated tick control methods, breeding tick resistant livestock strains, use of antitick vaccines, use of quarantine legislations, slaughtering infected animals, pasture spelling, use of fodder with high nutritional level to enable livestock withstand the stress caused by T&TBDs, and use of tick models to help select cost-effective strategies, are selectively used with little success. Some are rarely used while others are still either under development or unknown to the end users (rural livestock farmers). People education and awareness campaign programmes and events like in the case of Lyme disease vector ticks in Europe and North America have never been effectively, efficiently, and extensively planned and conducted for the case of livestock ticks. The sustainability of tick control and management interventions are always marred with a variety of problems depending on formulated tick control and management strategies and policies, scientific opinions of stakeholders, and government legislation and political will of involved countries [33–35]. Shortcomings in certain tick control and management interventions such as partial control of* Boophilus microplus* by vaccination [29] and technical difficulties in execution of large-scale tick control and management interventions [36, 37] are among the obstacles that have continued to undermine successful T&TBDs control operations even when using alternative methods available. Socioeconomic constraints, political strife, lack of trained personnel, and a poor infrastructure are major contributory factors to unsuccessful tick control and management interventions [22, 23, 33]. Incorrect administration of developed tick control tools and their continued failure to be efficient and effective are some of the obstacles reported during T&TBDs control operations [37, 38]. However, enforcement of appropriate legislation and good management of developed tick control interventions have, in the past, scored successes in some areas [22].

The failure of many developed tick control and management interventions has not been only due to the above problems but was also caused by the manner in which they are planned, developed, implemented, monitored, and evaluated [22]. For instance, many programmes are generated without taking into account the existing traditional livestock farming systems and conditions, production objectives, priorities, resource base, and technical-know-how of rural livestock farmers [39]. Many top-down tick control interventions rely solely on researchers' professional expertise to identify research problems and draw up research agendas and priorities without consulting and involving the end users' (rural livestock farmers) cultural values, social practices, and opinion [40, 41]. Developing safe, economically affordable, user friendly, easily adaptable and accessible, environmentally friendly, and community-driven interventions well suited to local conditions and specific to rural livestock communities can be highly successful and would be desirable [21, 42]. Community-specific and locally available antitick plants [43, 44] and other none botanical antitick ethnopractices and agents [43, 45] are promising but neglected strategic alternatives in tick control and management programmes. Although work on these plants in 1980s and 1990s revealed a resource with great potentials (antitick plants with toxic, repellent, attractant, antifeedant, and growth regulating properties) [46], this strategy has remained neglected and unexploited [47]. Most important is the fact that these plants are holistic in action [39, 48] and, therefore, have many positive values to offer to rural livestock farmers [49]. Such a multipurpose intervention best fits the recent ascendancy of individual livestock owners as the main players in tick control programmes, particularly following the withdrawal of most African government agencies in mid-1980s [50, 51]. This paper reviews the potential role and contribution of ethnoknowledge on ectoparasite control with a special focus on ethnobotanical acaricides in integrated tick control and management programmes. The review constitutes a consolidated database of previously used or mentioned plants with antitick properties, including antitick knowledge reported in non-peer-reviewed publications. Only some of these plants so far have been experimentally evaluated and assessed for their acaricidal/ectoparasiticidal activity.

## 2. An Overview of Sources of Information on Plants with Effects on Livestock Ticks

This section describes and discusses a varied number of sources and methods used to access information on plants and plant products with effects on livestock ticks worldwide. The identification of sources of information of ethnobotany of veterinary importance, local veterinarians, paraveterinarians, and agricultural extension officers responsible for providing extension services to livestock farmers in Kenya were accessed and discussions held. Local livestock traders and dealers, as well as individual livestock farmers, contributed their knowledge of ethnoveterinary medicine based on their professional and economic activities. Local ethnopractitioners, including general traditional healers/herbalists, diviners, curse detectors, and specialized medicine men and women formed a particular special subset of knowledgeable people from whom information was accessed. Secondary data were key source of information for this particular study and provided a very important source of leading ethnobotanical information of veterinary importance. Sources of secondary data included the following: local veterinary offices, herbaria libraries, and websites/URLs and databases of various relevant research institutions and centres worldwide. All these groups were consulted because each was associated with a specific aspect of useful ethnobotanical knowledge relevant to the study.

## 3. Ethnoknowledge of Ectoparasite Control and Management

This is a culture-bound knowledge system found within ethnoveterinary medicine, which has evolved concurrently with human ethnomedicine [52–54]. Understanding ethnopractices involved in ectoparasite control and management is necessary in the verification processes so that any research effort is not wasted on chemical analysis of plants that are used for culturally specific reasons [55]. For example, a study conducted in Trinidad and Tobago on a wide range of ethnoveterinary plants [55], through cultural comparative analysis with reference to the existing literature and by a method of nonexperimental validation of herbal medicines/products, resulted in the following list of 13 cultural plants that were selected for use in integrated control and management of the cattle ticks,* Boophilus microplus* and* Amblyomma cajennense* (locally known as “Garrapat” and “Cayenne,” resp.), the dog tick,* R. sanguineus*, and mites [56] (see Table 1).

Ancient communities particularly those that practiced pastoralism understood the concept of contagion and vector-borne disease of livestock involving ectoparasites [39]. They had control measures put in place to help avert economic losses incurred due to these parasites [39, 59–61]. Although most of the pertinent literature is anecdotal, several recent studies have shown that wild animals naturally select certain plant species and use them for management of ectoparasites infesting their respective places of residence [62]. Virtually all ancient stock raising societies had ways to control and manage livestock ectoparasites that plagued their animals. For instance, Nigerian Fulani correctly observed that* Sammore* (Trypanosomiasis) was spread by tsetse fly bites [63] and used a variety of traditional methods to control them [39]. The East African pastoralist communities knew long before the advent and introduction of western veterinary science that redwater scourge in cattle and heartwater bane in sheep were caused by the bite of grass ticks, which infested the grazing grounds [48]. Similar beliefs implicating ticks as vectors of deadly livestock diseases were held by Somali [61], Dinka [64], Fulani [60], and South African early settlers [39]. Other ectoparasites well known to ancient stock raisers as sources of livestock diseases include the following: biting flies, fleas, lice, and mites (causative agents of livestock mange/scabies) [48]. Control and management of these livestock ectoparasites have been summarized in Table 2. Within the phylum Arthropoda, one ethnoremedy technique or practice could or can be used for controlling and managing more than one ectoparasite [48]. For instance,* Nicotiana tabacum* decoction/suspension was used by the Gikuyu women in Kenya to control ticks, by the Nigerians to control biting flies, by the Bulgarian nomads and the Andeans to control mites, mange, and scabies, and by the Samburu in Kenya to control leeches (Table 2). This shows the holistic nature of ethnobotanical remedies (natural bioactive compounds from plants), the much needed technology [65] suitable for deployment in integrated pest management by resource-poor livestock farmers [22].

## 4. State of Knowledge on Ethnobotanicals That Affect Livestock Ticks 

The world is endowed with a vast diversity of plants ranging from microbial organisms, terrestrial plants, to marine flora. Throughout the evolutionary history, these plants have been an important resource for human and animal community. Besides being a source of food, several of these plants have been investigated for medicinal and pesticidal activities, while others are being explored for plant and arthropod growth regulators, allelochemicals, arthropod antifeedant, repellent, and toxicity. Some of these have potential for serving vital prototypes for structure optimization chemical technology [92].

Today, there is a growing appreciation of the value of ethnobotanical veterinary knowledge (EVK) among western trained professionals and periurban communities around the world [18, 48, 67, 93–100]. Because of great interest in and acceptance of EVK as alternative for disease control, ethnoveterinary research and development (ER&D) of which antitick ethnoknowledge is an integral part, has become a fertile area of technology development [101]. This knowledge has proved valuable not only to those who depend on it in their daily lives (mainly pastoralists) but also to modern industries and agriculture as well [48, 97, 102]. Many widely used products, such as plant-derived pharmaceuticals, acaricides, nutraceuticals, functional foods, hormones, pesticides, herbicides, insecticides, aromatics, and cosmetics, originate from traditional knowledge as their source [102–104]. EVK provides hope for economically impoverished local communities whose livelihood is livestock-dependent [48, 94, 95, 99, 105, 106]. Applications and studies of EVK have put more emphasis on the control and management of livestock ectoparasites (Table 2). The current review focuses on traditional control and management of livestock ticks, with a special focus on ethnobotanical substances that affect and modify tick behaviours.

Antitick ethnobotanical knowledge has its origin too rooted in trial and error traditional practices of ancient people. This was an early attempt to free livestock from ticks and other related arthropod pests. Before the advent of modern acaricides, a number of them being defunct now, ancient communities had developed a number of ways of controlling and managing arthropod pests of livestock, ticks inclusive (Table 2). Today, these ancient practices continue to yield interesting and potentially useful research leading to scientific discovery of much needed acaricides. For instance, the demonstration of repellent properties of molasses grass,* Melinis minutiflora* Beauv. (Poaceae) against* Margaropus annulatus australis* in South America by Menendenz (1924) [107], was much guided by ethnoknowledge of indigenous communities. Following this initial understanding, ten years later, De Jesus (cited by Thompson and coworkers [108]) was able to demonstrate too that* M. minutiflora* had deleterious effects on ticks,* Boophilus australis*. After 43 years of De Jesus observation, Thompson and coworkers [108] too observed that molasses grass together with gamba grass,* Andropogon gayanus* Kunth (Poaceae), showed tick-repellent properties in pastures in Colombia. In Africa, there was not any such report on antitick plants until the early 1990s when Dipeolu and coworkers (1992) [109] reported the acaricidal potential of an African spider flower,* Gynandropsis gynandra* (L.) Briq. (Capparaceae). This therefore implies that, up to date, meaningful research and development on antitick plants are still lacking. However, based on the compilation of literature on the said botanicals (Table 3), there is much evidence that some plants contain compounds that affect and modify tick behaviour (referring to botanicals with ability to kill, repel, immobilize, and affect tick fecundity and growth). For instance, plants of the genus* Stylosanthes*, apart from being a tropical forage legume, have shown great potentials for immobilizing and killing tick larvae [110–115]. Pastures with* M. minutiflora* have been shown to affect ticks by reducing their ability to live for longer [107, 108], thus an implication for the presence of some compounds that may induce tick mortality. In East Africa,* G. gynandra *has been shown to have antitick properties in pasture lands [44, 109]. In Kenya, a number of plants have been shown to affect and modify tick behaviours, particularly with repellency potentials. For instance,* Commiphora erythraea* and* C. myrrh* [116]; gum of* C. holtziana* [117];* Cleome monophylla* [118];* Ocimum suave* [119]; and* G. gynandra* [120] have been demonstrated to contain essential oils which have repellent components against* Rhipicephalus appendiculatus*. Various tribes in Kenya have traditionally used different plant-derived materials to control ticks. For instance, Gikuyu people (women) have used leaves of tobacco,* Nicotiana tabacum* [121]; Somalis have used gum resins and myrrh of* Commiphora* spp.; Luos in Nyanza have used leaves of* Aloe* spp.; and the Turkana people use leaves of* Acalypha fruticosa* to control and manage livestock ticks [67]. In Kenya, some tribes grow neem* (Azadirachta indica)* and or other plants they consider to have repellence property such as* Euphorbia balsamifera, Sesbania sesban*, and* Cissus purpurea* near animal housing for the purpose of repelling ticks [67]. Although a number of these plants have been documented in some local communities and some scientifically evaluated, still rural livestock farmers, extension workers, and other relevant stakeholders cannot put them on beneficial and wider utilization due to lack of information on their formulation, standardization, optimal concentration, and application regimes. This has been partly attributed to lack of knowledge on the identity and description of plants' active substances, which affect and modify tick behaviours.

This review provides a list of plants (Table 3) with leading information on how they affect and modify the behaviours of various livestock tick species under different geographical and environmental conditions. In addition, the review gives a basis for scientific research on the development of natural products for tick control and management and tick-borne diseases.

## 5. Plant Species with Prospects for Tick Control and Management in Africa: Opportunities and Threats

Table 3 presents a list of enumerated 209 plant species with their correct botanical and common names arranged in the first column, followed by family names in the second column, plant part(s) used in the third column, application form and action in the fourth column, location in the in the fifth column, and references made in the last column. Practically, all these plants have been “discovered” through information derived about their use in traditional medicine sense. The plant species showed a wide range of bioactive properties on different livestock tick species with 40% of 210 reported research on ethnobotanical acaricides being conducted in Africa, which is the most affected continent with tick infestation and tick-borne diseases, and henceforth, the associated socioeconomic losses [23]. This provides for the feasibility of controlling livestock ticks in Africa with plants and their products as components of an integrated tick control and management programme. This feasibility is justified by numerous scientific and socioanthropological reports of surveys and laboratory studies on African plants with effects on livestock ticks [104, 126, 127, 129, 149, 151, 165, 166, 199, 204–210]. A number of plants that have been conventionally examined for acaricidal properties include* Melinis minutiflora* [119, 211],* Commiphora erythraea* and* C. myrrh* [116],* Ocimum suave* [179],* Margaritaria discoidea* [174],* Tephrosia vogelii* [199],* Azadirachta indica* [46, 141, 142, 212],* Nicotiana tabacum* [43],* Gynandropsis gynandra* [44, 109, 120],* Euphorbia candelabrum* [140], and* Ageratum houstonianum* [126]. Other many African plant species with potential for use in tick control and management programmes have been documented and recorded during the many ethnoveterinary survey studies conducted in local African communities [47, 67, 104, 117, 127, 151, 153, 165, 166, 198, 199, 208]. These surveys play a key role in providing a common ground for conventionally trained researchers and ethnopractitioners (rural resource-poor livestock farmers) to meet and interact as equal partners in the war against T&TBDs. However, this is not the case. The rural resource-poor livestock farmers feel that they are being short-changed and exploited during such useful interactions and that their ethnoknowledge may be patented by researchers without their consent and earning any benefits accruing from it on which their entire livelihood is dependent [213]. Bridging this gap of mistrust between ethnopractitioners and researchers poses great challenges and, nevertheless, it remains the biggest stumbling block and threat to any fruitful development of ethnoknowledge in Africa.

Extracts from plants in Table 3 have been shown to possess strong acaricidal and/or tick-repellent bioactivities. Some of the plant extracts are capable of affecting and modifying tick feeding behaviours, molting processes, fecundity, and viability of eggs, and so forth. Some of these plants are suitable forage and due to secondary metabolites they secrete as viscous, adhesive, odorous, or toxic substances; they have been found to be capable of repelling, trapping, and killing different host-seeking tick species [110, 131, 136, 206, 209, 214]. Some plants possess hairs (trichomes) that prevent ticks from climbing to the top in order to attain a suitable posture for attaching to any passing suitable host animal [44]. The existence of plants with these multiple bioactive properties in Africa offers challenges and opportunities to save African countries the high costs for importing acaricides and to replace those rendered unusable with tick resistance by critically considering the possibilities of using indigenous African plants as sources of acaricides [209]. These many bioactive properties indicate that the plants or the resultant herbal products can be integrated in various combinations for the control and management of different tick species especially by rural resource-poor livestock farmers at affordable or no cost at all. For instance, antitick grasses such as* M. minutiflora* and shrubs such as* Gynandropsis gynandra* and* Ocimum suave* could be planted in pastures to repel, immobilize, and kill ticks, thus reducing the number of ticks attaching on cattle. This option, in combination with tick attractant shrub,* Acalypha fruticosa* [122], can be used by rural resource-poor livestock farmers to develop livestock pastures free from tick infestation. Additionally, antitick plants, which serve as forage can also be used in pasture-spelling systems with species suitable for tropical and subtropical regions in an integrated tick management (ITM) control system [33, 108, 132, 136, 206, 209, 214]. On the other hand, botanical extracts (e.g., neem, Kupetaba,* G. gynandra, O. suave*,* M. minutiflora*, and* M. discoidea*) would be applied directly on cattle to repel, to disrupt feeding and molting behavioural processes, and to kill the on-host ticks without ticks developing resistance and extracts causing any side effects and environmental pollution. Other traditional methods of tick control and management, for example, hand deticking and intergrazing (grazing sheep ahead of cattle), could also be integrated into the above-mentioned management system options. These control strategies would be effective and affordable by many rural resource-poor livestock farmers in Africa and elsewhere since the seeds of grasses and other antitick plants are inexpensive, locally available, and easily accessible and the technology is simple and easy to apply without the assistance of an external personnel. The technology would also be easily adopted by rural resource-poor livestock farmers since most of them are familiar with the plant materials and are already using one or more of these traditional methods for tick control and management on their farms or within their homesteads (Author's experience in Bukusu and Wanga communities in western Kenya).

Unfortunately, in Africa, there are no commercial uses of these ethnobotanicals and awareness campaigns for their integration into tick control and management systems. The application of ethnobotanicals for tick control and management is still confined to individual rural resource-poor livestock farmers or ethnopractitioners and researchers in their respective institutions. Researchers and rural resource-poor livestock farmers are still independent and closed, each group working on its own without letting the information to benefit either side with critical and open mind. The plants in Table 3 and others yet to be discovered from African communities will only be of value to and benefit African livestock industry if the gap of mistrust between ethnopractitioners and researchers is amicably bridged and the active participation of natural custodians of biodiversity and ethnopractitioners (rural resource-poor livestock farmers) of valuable knowledge is guaranteed in the generation of research focusing on screening programmes dealing with the isolation of bioactive principles and the development of new livestock ectoparasiticides [213].

## 6. Potential and Future Prospects for the Use of Antitick Plants in Tick Control and Management

From the foregoing, the list of antitick plants or plants with acaricidal properties shown in Tables 2 and 3 is not yet exhaustive as more plant species with such activities may be discovered. Efforts to promote hands-on use of antitick plants at community level have clearly increased as signaled by a worldwide documentation of the plants (Tables 2 and 3) and many more studies being conducted on field surveys and trials [58, 109, 122, 129, 149] and laboratory tests [73, 96, 215–220]. The ongoing workshops, community-based training seminars, and novel projects on antitick plants at the Intermediate Technology Development Group-Eastern Africa (ITDG-EA), an international nongovernmental organization [129], are other promising proliferation signals in this area. The many ethnoveterinary projects and proposals identified at the November 1997 Pune conference in India signaled a worldwide recognition and appreciation of the potential of EVK in the development of livestock industry [221]. This recent and renewed interest in herbal products as a reemerging animal health aid has been fueled by the rising costs of livelihood-dependent conventional products, their adverse side effects, genetic selection for resistance by target organisms, and continued unavailability in time and quantity to meet the health needs of the growing population. The situation has been exacerbated by continued lack of trained veterinary personnel in remote rural areas and bioprospecting and biopiracy of new herbal products by researchers mainly from industrialized societies [213, 222]. As the focus increasingly concentrates on exploitation of herbal products, the stakeholders should be made aware of the risks involved in and limitations of using these products.

Side effects and toxic reactions to herbal products are considered rare [223–225], albeit their existence being as old as human history [226]. There are claims that toxic effects of herbal products are often not the fault of the herb itself per se but are caused by products containing misidentified plants or contaminants such as bacteria and heavy metals during the preparation of the remedy [227, 228]. Allergic reactions to herbal products following contact during preparation and or application can occur, as with any other plant material. Toxic problems associated with particular plants or product types are well documented and understood [229–232]. Some plants are inherently toxic, containing naturally occurring toxins, often with cytotoxic or carcinogenic effects. While the identities of the more common toxic plants are generally known, at least to plant phytochemists, older herbal texts may not reflect this knowledge [224].

However, risks involved in using herbal products originate from three main groups of causes. The first group of cause is intrinsic toxicity of several plants that has been outlined since the 1960s [228, 231]. For instance, active ingredient in the antitick and mange castor-oil plant* (Ricinus communis)*, ricin, is very poisonous and provides an example of an EVK agent that must be handled with care while applying to the animal, just like conventional acaricides [233, 234]. The second cause of risks involves herbal products responsible for many dermatitis, psychological, and neurological side effects. While the third one is a result of inconsistence prescription and qualitative and quantitative measures due to ethnicity, culture, picking time, stage of growth, processing, storage, place, and altitude of growth and pollution by other pesticides, and so forth. Other risks include the following: (a) contamination by other plants, botanical confusion, and confusion between two almost similar names such as* Stephania tetrandra* and* Aristolochia fangchi* which are, in Chinese, “Han Fang Ji” and “Gang Fang Ji,” respectively [231, 235–239]. The confusion may also be induced by (a) same vernacular name for two species, for example, “Copalchi” refers to* Coutarea latiflora* (not toxic) and* Croton niveus* (toxic), (b) adulteration by allopathic drugs, (c) circumstances of use, this may result in misuse of herbal products or excessive drenching of animals at times, (d) many unknown factors related to the affected animals' habits/history, and (e) uncontrolled delivery of herbal products via Internet [228]. Some herbal products contain phytochemicals that have strong effects on the animals' body as part of their therapeutic action, that is, purgatives [240]. Highly purified or isolated extracts of plants, such as essential oils or other concentrated isolates, may have markedly different effects on the body or may even be quite toxic compared to less refined extracts of the same herb [241, 242]. Dose-related toxicity is of particular concern with any potent herbal product. As with all acaricides, following the recommended dose guidelines included with all herbal products would be a first line of defense against overdose.

In practice, EVK is not without disadvantages and limitations. Certain particular EVK application methods are often very much localised, harmful, and unhygienic and the scope for their further dissemination is limited. The EVK cures are variable in their effectiveness according to season, method of preparation, ethnicity, person's own experience, and so forth and very few plant products have been validated in the same way in which synthetic drugs must be validated. While some remedies are just inconvenient to prepare or use. From a technical standpoint, some plant products are totally ineffective and acute cases have very little if not nothing to benefit from EVK [234, 243, 244]. One of the most limiting factors of ethnobotanical acaricides is that they are not always practical on a large scale and dosages are uncertain and remedies are not standardized since the concentration of a critical ingredient in a plant often varies from one location to another and from one season to another. A particular EVK method may require considerable amounts of leaves, seeds, or even roots, which might not be possible to get. Further, certain plants are available only at some times of the year and the resource base is ever deteriorating, making ingredients unavailable for preparing products [234].

Although EVK has its limitations, there is tremendous scope for its use in interventions either singly or being integrated with scientific knowledge [245]. EVK practices are often cheap, time-tested, environment-friendly and safe, cost-effective, readily available, location-specific, and based on familiar local resources and strength. Currently, a large segment of the world's livestock population is still dependent on traditional knowledge and ethnoveterinary practices for its healthcare (more than 80% of the world population relies on it). Many of these ethnopractices offer viable alternatives to conventional western-style animal healthcare and are especially convenient to use and relevant in developing countries with limited financial resources. Local livestock keepers are already familiar with the plant products, which have been effectively and efficiently used over many generations and withstood the test of time. Most of the plant products are freely available or at a cost in proportion to the value of the animal to be treated and are easily administered, usually topically or orally. But unfortunately these ethnopractices are little documented in some cases and increasingly lost through poor storage means and their passage from generation to generation by word of mouth [246]. It is necessary therefore to have extensive documentation, campaign awareness, evaluation, and validation of the EVK and make it more homogeneous, more efficient, less mysterious, and more profitable to holders and users [219]. In addition, an interdisciplinary approach is essential because of the multiplicity of factors and a wide spectrum of techniques and insights (validation, enterprise, environment, health care delivery, public health, education, socioeconomics, sociocultural, networking, planning, and clear policies) involved in EVK [48, 101]. There is need therefore for protection and conservation of EVK genetic resources for sustainable utilization and development [99].

With regard to the control and management of ticks and tick-borne diseases using antitick plants, future field and laboratory experiences should target and focus on, at least, the following points:

- Specific target tick species and or related arthropod species that are concerned, namely, the genera* Amblyomma*,* Haemaphysalis, Rhipicephalus, Boophilus, Hyalomma, Dermacentor* and* Ixodes*; mites; biting insects/livestock biting flies; lice; fleas, and so forth
- Whole plant or plant parts/products to be used, namely, barks, leaves, flowers, buds, roots, stems, fruits, seeds, bulbs, juice, latex, gum, sap, and so forth
- The active compounds produced by the plants as well as methods of extraction, isolation, and formulation
- Other products to be associated and integrated with the chosen plant parts' products, namely, soot, salt, soap, soil, charcoal, ash, smoke, dew, any ritual performed, and so forth
- Application form, namely, drinking in water, drenching, decoction, infusion, pulverisation, dust, powdered, bolus, paste, juice, poultice, fomentation, compress, rubbing, pour-on, fumigation, hanging bouquet, steaming, and so forth
- Application rate that is the amount of the preparation to apply and the rate of application
- Action being toxic, repellent, acaricidal, antifeedant, antiovipositant, acute and residual effects, and so forth
- Collection and storage of antitick plants by sustainable harvesting of the plants, protection and conservation of the plants, and preservation of harvested and prepared materials
- Simple technologies of processing and applying the preparations, familiar to local resource livestock farmers
- Side effects of the preparations being short- and long-term effects
- Whether or not a given preparation is safe for human health

However, scientific validation of promising antitick ethnobotanicals on their efficacy and formulation of packages easily handled by local communities is necessary for a significantly increased use of such remedies, as they will be more feasible, and the results obtained would be more comparable [99]. Consequently, the value of antitick ethnobotanicals will become more easily recognized and understood by professionals from all walks of life in livestock health. However, even when funding for such work is available the researcher is faced with a level of variability that virtually defies control as required in traditional scientific experiments, for example, plant species, time of year of harvesting and growing, growing conditions, method of collection, storage, preparation, and administration [243]. This, however, makes herbal products very unique and thus very difficult to standardize and bring them at the same level as conventional products; hence the term* complementary* fits them best in their differentiation with synthetic products.

## 7. Conclusion

The antitick ethnobotanical plants offer great potentials in tick control programmes and management strategies. The traditional ways of using such antitick plants in the community can overcome many obstacles such as cost-related production problems, formulation, availability, accessibility, and environmental pollution and contamination. Moreover, their applications are simple and could be used by local communities with minimal external help. In addition, ethnobotanicals are biodegradable, holistic and additive, synergetic, and nutritive in their action and, because of these properties, they do not allow the development of resistance problems like their synthetic counterparts. However, little attention has been given to assess the effectiveness and efficacy of antitick ethnobotanicals in an integrated tick management programmes. For this to be realized, both at local and at global levels, there is need to evaluate the traditional knowledge and explain its scientific rationale where possible with a view to ascertaining safety and preventing abuse so that the “best bets” can be identified for integration into livestock health primary delivery systems. The effectiveness of these “best bets” ethnopractices can be greatest if appropriately blended with conventional technologies and active involvement of the livestock-holding communities. Based on findings of this review article, herbal products will seemingly play a greater role in animal health industry than ever before in this millennium. The impact of this role will be greatest if the active participation of such natural custodians and ethnopractitioners of valuable knowledge is guaranteed in the generation of research focusing on screening programmes dealing with the isolation of bioactive principles and the development of new livestock ectoparasiticides [213].

## Figures and Tables

**Table 1:** Plants used in the prevention, control, and management of ticks and mites of livestock in Trinidad and Tobago.

Scientific name	Family	Plant part used	Known active and other components
*Azadirachta indica*	Meliaceae	Leaves	Limonoids, azadirachtin, salannin, deacetyl-azadirachtin, and meliantriol
*Cedrela odorata*	Meliaceae	Leaves	—
*Cordia curassavica*	Boraginaceae	Leaves	Phenols and terpenoid quinones
*Eclipta alba*	Compositae	Plant tops	Polyacetylenes nicotine
*Mammea americana*	Guttiferae	Seeds	Mammein
*Manilkara zapota*	Sapotaceae	Seeds	HCN, sapotin, and saponin
*Momordica charantia*	Cucurbitaceae	Vine	—
*Musa species*	Musaceae	Stem juice	Caprylic acid and 5-hydroxy-tryptamine
*Nicotiana tabacum*	Solanaceae	Leaves	Nicotine
*Petiveria alliacea*	Phytolaccaceae	Leaves	—
*Pouteria sapota*	Sapotaceae	Seeds	Amygdalin
*Renealmia alpinia*	Zingiberaceae	Leaves	—
*Scoparia dulcis*	Scrophulariaceae	Plant tops	—

*Note*. Some of these plants were among the 43 plant species evaluated in Jamaica, whose crude ethanol extracts of the leaves for pesticidal effects on the engorged cattle tick, *Boophilus microplus*, were determined [57, 58]. Their acaricidal indices (AI) for the crude plant extracts ranged from 50 to 100. Among the plants studied were *Momordica charantia* (AI = 71), *Azadirachta indica* (AI = 68), and *Petiveria alliacea* (AI = 66).

**Table 2:** Traditional prevention, control, and management of livestock ectoparasites by native and local communities.

Target livestock ectoparasite(s)	A description of traditional remedy	References
Small red flies	(i) Setting smudge fires in the sheds (ii) Rubbing kerosene and other substances on the animals	[66]

Tick infestation in livestock populations	Piercing ticks with a needle or a blade	[61, 67]
	(i) Feeding salty plants to animals so that the ticks can fall off (ii) Every morning, picking off and burning any ticks they find on their animals (iii) Placing thorn bushes on infested places so that camels should not roll on them	
	(i) Pounding 5 leaves of *Aloe broomii *and mixing with 300 ml of paraffin oil and 2 handfuls of kitchen ash to make a paste for smearing on the infested parts of the animal (ii) Use of hagar, *Commiphora erythraea*, or damaji, *C. incisa*, by Gabbra and Somali in Kenya (iii) Smearing leaf paste of *eteteleit*, *Acalypha fruticosa*, by the Turkana in Kenya (iv) Bathing animals with salt solution (~100 g of salt dissolved in 1 litre of water) (v) Rubbing old engine oil on infested areas of the animal's body (vi) Allowing animals to wallow in shallow, muddy pools (vii) Using animal quarantine techniques to keep infected stock away from noninfected one (viii) Removal of weeds and bushes from livestock housing (ix) Predation of ticks by birds-keeping chickens in and around the animal housing (x) Raising neem, *Azadirachta indica*, or other tick-repellent plants near animal housing	[67]
	Use of tobacco, *Nicotiana tabacum*, by the Gikuyu in Kenya	[45, 67]
	(i) Avoiding infested pastures, fodder, shade trees, and cool places which favour ticks' survival (ii) Burning of livestock pastures and tick-infested manure in the sheds	[61, 67]
	Drenching animals with a mixture of salt and six ground fruits by the Twareg	[68]
	Handpicking of ticks during milking by Fulani women and children in Burkina Faso	[60]
	Use of toasted *maych'a* leaves to drive out ear ticks in herd animals in Peruvian Andes	[69]
	A herbal preparation pestban in control of ectoparasites in household pets and domesticated animals in India	[70, 71]
	Acaricidal activity of the combination of plant crude extracts to tropical cattle ticks *(Boophilus microplus)* in India	[72, 73]
	A herbal ectoparasiticide AV/EPP/14 against lice and tick infestation on buffalo and cattle in India	[74]

Livestock insects	Lighting smudge fires beside resting buffalo, cattle, Amerindian horses, Siberian reindeer, and Andean guinea pigs	[59, 66, 75, 76]

Livestock pests	Andeans burnt old tires in corrals	[39]
	In Andean region, corrals were sprinkled with lime, kerosene, or creosote on affected animals	[77]
	Seasonal burning of rangelands used for grazing in Andes and Africa	[39]
	A herbal preparation pestban in control of ectoparasites in household pets and domesticated animals	[70, 71]

Parasitic Insects	Fumigation of animal quarters and camps with herbs by Nigerian Pastoralists	[63, 71]

Tsetse flies	Washing cattle with an infusion of *Sesbania aculeata*, ointments, dust, and tobacco by Nigerian pastoralists	[63]
	(i) Bathing animals' body with emulsion made from roots of *Cissus purpurea* (ii) Bathing animals' body with emulsion made from leaves of *Sesbania sesban* in Kenya (iii) Smearing the oil of neem, *Azadirachta indica*, seed kernels on animals' bodies (iv) Smearing the latex of *Euphorbia balsamifera* on the bodies of affected animals	[67]

Livestock biting flies	(i) Nigerian horses bathed with fly-repellent liquids (ii) Yoruba employed soap mixed with graded roots of the violet tree (iii) Northern Nigerians applied tobacco-based ointment	[75]
	Venezuelan fly repellent was a wash of squash-leaf juice	[78]
	(i) Washing animals with a suspension of fresh root of *anthata* of Gabbra in Kenya (ii) Smoke from burning cow dung drove the flies away (iii) Position livestock sheds to allow wind to blow flies away and avoid flies-infested areas	[67]

Livestock fleas and lice	A herbal ectoparasiticide AV/EPP/14 against lice and tick infestation on buffalo and cattle in India	[74]
	(i) Sweep livestock sheds with brooms of *Tagetes minuta *(Mexican marigold) or desert rose, *Adenium obesum, *or burn *T. minuta* leaves in sheds and wash cattle with *A. obesum * (ii) Sprinkling Magadi soda powder in sheds or wash animals with suspension of *A*.* obesum * (iii) Wash animals with suspension of *Aloe* spp. *(A. secundiflora, A. kedongensis, and A. lateritia)*	[67, 70, 71, 74]

Livestock lice per se	(i) Wash animals with suspension of *Aloe* spp. and sisal, *Agave sisalana* (ii) Smearing cattle with mixtures of fruits of *adekelait* and *akej etom* of Turkana in Kenya (iii) Wash animals with suspension of garlic, *Allium sativum* (iv) Use of eucalyptus, blue gum, *Eucalyptus *spp. (vi) Rubbing camel's urine on the infested animals' skins (vii) Smearing a mixture of camel's urine and salty soil on animal's skin (viii) Smearing cow dung on the infested body areas of the animals (ix) Smearing goats', donkeys', sheep's, and camel bones' fat over the animals' bodies (x) Washing affected animals with natural salty water (xi) Rubbing a paste of clay (*dhoobo* in Somali) on the affected animals (xii) Shaving camels' hair and rubbing skin with a mixture of camel's urine and salty soil (xiii) A herbal preparation pestban in control of ectoparasites in household pets and domesticated animals (xiv) A herbal ectoparasiticide AV/EPP/14 against lice and tick infestation on buffalo and cattle	

Nasal bots	(i) Putting in nostril a suspension of root of *abach* by the Turkana of Kenya (ii) Keeping animals away from thickets in the rainy season (iii) Giving animals drinking water at salty sources to enable them expel the larvae (iv) Putting in nostril a suspension of root of *entulelei(Solanum incanum)* or *olgrigiri (Acacia brevispica)* by the Maasai of Kenya (v) Putting in nostril juice from ripe fruits of sodom apple, *S. incanum*, by Kamba people (vi) Putting in nostril a teaspoonful of root suspension of *Ingalayioi* (*Cucumis* sp.) by the Samburu of Kenya (vii) Putting sheep milk into nostrils of affected animals to make them sneeze out maggots (viii) Passing in nostril smoke of the bark of *Ingeriyioi* or *Imasei (Tarenna graveolens)* by the Samburu of Kenya	

Leeches	(i) Use of tobacco, *Nicotiana tabacum*, and *Saali le tim* suspension by Samburu in Kenya (ii) Avoiding leech-infested areas and physically removing attached leeches from animals	[67]

Swine ectoparasites	Banana leaves and an extract of garlic in Central Brazil	[79]

Chicken lice	A wash of vinegar and lemon juice by Andean people	[39]

Skin sores of cattle	Dust with the powdered dung of ostriches and hyenas by the Neur	[59]

Lice and mites	A herbal preparation pestban in control of ectoparasites in household pets and domesticated animals	[70, 71]
	Andean stockowners used *barbasco*	[80]

Mange/scabies caused by ectoparasitic mites	Root of *Rumex patientia* L.	[81]
	Latex from *Euphorbia somaliensis* or camel urine	[82]
	An infusion of *Iphiona rotundifolia* plant	[61]
	Rubbing rhubarb and caustics into the mange lesions in China	[83]
	Rubbing a decoction of tobacco leaves into the mange lesions by Bulgarian nomads	[39]
	Topical application of wild tobacco leaves and black soap by the Andeans	
	Andean *muna (Minthostachys andina) *and tarwi plant *(Lupinus mutabilis)* provide treatment for mange	
	In France, milk, vinegar, olive oil, lard, ashes, soot, sulphur, turpentine, crankcase oil, and mineral waters were administered as pomades, plasters, lotions, drenches, or feeds.	[39, 84]
	(i) Washing animals with a suspension of *(Oldarakwa)* pencil cedar, *Juniperus procera* (ii) Drenching and smearing animals with a suspension of ash made from branches of *Ng'adapala (Dobera glabra) *by Kenyan pastoralists (Turkana) (iii) Keeping animals' pens and surroundings clean and dry (iv) Using animal quarantine techniques to keep infected stock away from noninfected one (v) Smearing motor oil and or sesame oil on the affected areas of the animals (vi) Turkana people use a suspension made from stems of *eligoi* to drench and wash animals (vii) Use of *Iparaa*, *Euphorbia* sp., by Samburu of Kenya (viii) Powdered charcoal of *esekon* (toothbrush tree), *Salvadora persica* mixed with 1 litre of ghee to make a paste for topical application by the Turkana	[67]

Alpaca mange (caused by mites)	Use of pig fat, rancid camelid grease, boiling-hot lard, rancid urine, sulphur, stove ash, soot from earthen cookpots, masticated coca leaf, old motor oil, and battery acid	[81, 85–89]

Ruminant ectoparasites	Water of tarwi plant, *L. mutabilis*, combined with ash of burnt cattle manure	[69, 90]
	Compounds of tarwi plant, *L. mutabilis*, and other botanicals by Andean smallholders	[86, 91]

**Table 3:** An enumeration of plant species that have been documented in literature to contain compounds and/or active ingredients that have effects on livestock ticks worldwide.

Species name of plant (English name)	Family name of plant	Part(s) of plant used	Application form/action (effects) and target tick species	Place	Source/ Reference
*Acalypha fruticosa *Forssk. Var. *Villosa* Hutch	Euphorbiaceae (spurge family)	Leaf	Sediment smeared onto the ears as repellent against ticks by the Turkana people of Kenya (aqueous preparation) *eteteleit* (Turkana language)	Kenya	[67]
			As tick attractant as observed in the field and laboratory (Luo, *Abaki*)	Kenya	[122]

*Acorus calamus* L.	Araceae (arum/ginseng family)	Rhizome	Repellent (aqueous and alcohol extracts) against *Ixodes* spp.	USA	[18, 123, 124]

*Allium sativum* (Link.) Döll. (Garlic)	Alliaceae (onion family)	Leaf/bulb	Eat garlic pills, tick repellent against *Ixodes* spp.	Mecklenburg County, North Carolina, USA	[125]

*Ageratum houstonianum *P. Mill. (Blue Mink)	Compositae/Asteraceae (daisy/aster family)	Essential oil from flowers	Toxic to ticks *(Rhipicephalus lunulatus)* at LD_50_ = 0.06653 *µ*l/cm^2^ within 24 hrs.	Cameroon	[126]

*Aloe broomii* Schonl.	Xanthorrhoeaceae	Leaf	Boiling in water to make cattle dip and disinfectant. Oral leaf juice is made for cattle or for topical application	South Africa; Kenya	[67, 127]

*Aloe ferox* Mill. (cape aloe, bitter aloe, red aloe, and tap aloe)	Xanthorrhoeaceae	Leaf	Infusion had a strong dipping and topical toxicity effect against *Rhipicephalus appendiculatus* ticks	South Africa	[128]

*Aloe marlothii* Alwin Berger (mountain aloe or the flat-flowered aloe)	Xanthorrhoeaceae	Leaf	Dichloromethane extracts were repellent to *Rhipicephalus appendiculatus*	South Africa	[128]

*Aloe *spp.	Xanthorrhoeaceae	Leaf	Topical application of a paste of leaves, paraffin oil, and kitchen ash	Kenya	[67]

*Aloe secundiflora* Engl. (aloe)	Xanthorrhoeaceae	Whole plant	A concoction mixed with labia plant *(Psiadia punctulata)* to make an effective acaricide against brown ear tick *(Rhipicephalus appendiculatus)*, red-legged tick *(Rhipicephalus evertsii evertsi)*, *Boophilus decoloratus*, and bont tick (*Amblyomma *species)	Kenya (Samburu pastoralists) in Baragoi	[129]

*Andropogon gayanus* Kunth (bluestem, gamba, or llanero grass)	Poaceae or Gramineae (the grass family)	Whole plant	Toxic/repellent	South America, Mexico, Colombia	[108, 130–134]

*Annona squamosa *L. (sugar apple, custard apple, and sweetsop)	Annonaceae (custard apple family)	Leaf	Leaves rubbed over floors or placed in hens' nests to keep away vermin which includes ticks	India and Mexico	[135]

*Artocarpus altilis* (Parkinson) Fosberg (breadfruit)	Moraceae (fig/mulberry family)	Fresh leaf	Topical application of crude ethanol extracts/being toxic and inhabitant of oviposition and embryogenesis of *Boophilus microplus* Canst.	Jamaica	[58, 136, 137]

*Asclepias curassavica* L. (redhead)	Asclepiadaceae (milkweed family)	Fresh leaf	Topical application of crude ethanol extracts/being toxic and inhabitant of oviposition and embryogenesis of *Boophilus microplus* Canst.	Jamaica	[58]

*Artemisia absinthium* L. (wormwood)	Asteraceae	Whole plant	Essential oils from the plant have been shown to have acaricidal activity	Europe, Eastern North America	[137] http://www.florahealth.com/about_int.cfm?sub_link=Export

*Artemisia herba-alba* Asso (white wormwood)	Asteraceae	Aerial parts	Diethyl ether, ethyl acetate, hexane, and ethanol extracts showed toxicity against larvae of *Hyalomma dromedarii* Koch, 1844	Egypt	[138]

*Artemisia tridentata* Nutt. (big sagebrush)	Asteraceae	Leaves	Toxic to nymphal ticks (*Ixodes scapularis* (Say)) (LC50 = 0.180% wt : vol)	USA	[139]

*Artemisia monosperma* Del. (Tarragon)	Asteraceae	Aerial parts	Diethyl ether, ethyl acetate, hexane, and ethanol extracts showed toxicity against larvae of *Hyalomma dromedarii* Koch, 1844. Essential oils showed toxicity effects to the larvae of *H. dromedarii* and *Argas persicus* Oken, 1818, adults	Egypt	[138]

*Azadirachta indica *Adr. Juss. (neem tree)	Meliaceae (mahogany family)	Whole plant; leaf; fruit	Repellent/toxic/inhabitant of oviposition and embryogenesis of *Boophilus microplus* Canst.	Kenya Jamaica	[67, 140] [58, 136]
		Fruit	Oil extracts caused mortality of *Amblyomma variegatum* larvae	Nigeria	[141]
			Neem seed oil extracts caused mortality of *Hyalomma anatolicum excavatum* Koch larvae and malformation or deformities in developing ticks	Egypt	[142]
		Neem oil and azadirachtin EC formulation	At 2500 mg litre^−1^, azadirachtin caused significant reduction in feeding activity of larva *(Hyalomma dromedarii)*, prolonged the period for molting to nymphal stage, and caused 60% reduction in moltability. Contact and dipping LC_50_ values were >40.7 *µ*g cm^−2^ and >5000 mg litre^−1^, respectively.	Saudi Arabia	[143]
		Seed	Neem seed oil as an acaricide	India	[144]
		Seed	Neem seed oil as an acaricide against *Boophilus microplus*	India	[145, 146]
		Seed	Neem seed extracts as acaricide against *Boophilus microplus*	Colombia	[147]

*Bixa orellana *L. (annatto)	Bixaceae (achiote/annatto/lipstick tree family)	Fresh leaf	Topical application of crude ethanol extracts/being toxic and inhabitant of oviposition and embryogenesis of *Boophilus microplus*	Jamaica	[58]

*Blighia sapida* (ackee, akee, or achee)	Sapindaceae (soapberry family)	Fresh leaf	Topical application of crude ethanol extracts. Being toxic and inhabitant of oviposition and embryogenesis of *Boophilus microplus*	Jamaica	[58]

*Bocconia frutescens* L. (tree celandine/parrotweed/plume poppy)	Papaveraceae (poppy family)	Fresh leaf	Topical application of crude ethanol extracts. Being toxic and inhabitant of oviposition and embryogenesis of *Boophilus microplus*	Jamaica	[58]

*Bontia daphnoides *L. (kidney bush/white alling)	Myoporaceae (Lamiales)	Fresh leaf	Topical application of crude ethanol extracts. Being toxic and inhabitant of oviposition and embryogenesis of *Boophilus microplus*	Jamaica	[58]

*Boscia angustifolia* A. Rich	Capparidaceae	Aerial parts/oil	Repellency of their essential oil	Kenya	[104]

*Boscia mossambicensis* Klotzsch	Capparidaceae	Aerial parts/oil	Repellency of their essential oil	Kenya	[104]

*Brachiaria brizantha* (Hochst) Stapf cv. *Marandu* (marandu grass, surinam grass, signal grass, and Kenya sheep grass)	Gramineae	Whole plant	Antitick properties	South America, Brazil	[131]

*Brachiaria decumbens *(signal grass)	Grass family Panicoideae	Whole plant	Weak toxic/repellent	South America	[108]

*Cadaba farinosa *Forssk.	Capparidaceae	Aerial parts/oil	Repellency of their essential oil	Kenya	[104]

*Cadia purpurea* (G. Piccioli) Aiton	Caesalpiniaceae	Whole plant	A concoction mixed with *Olea europaea* subsp. *Cuspidata* (African olive tree) to make effective acaricide against brown ear tick *(Rhipicephalus appendiculatus)*, red-legged tick *(Rhipicephalus evertsii evertsii)*, *Boophilus decoloratus*, and bont tick (*Amblyomma *species)	Kenya (Samburu pastoralists) in Baragoi	[129]

*Calocedrus decurrens *(incense cedar and California post cedar)	Cupressaceae (cypress family)	Ground heartwood and leaves	Toxic to nymphal and larval ticks (*Ixodes scapularis* (Say)) (LC50 = 0.343 and 0.015% wt : vol, resp.)	USA	[139]

*Calotropis procera* (Ait) R. Br. (rooster tree, giant milkweed, and sodom apple)	Asclepiadaceae (milkweed family)	A cardiac glycosidal (cardenolide) extract	Contact and dipping LC_50_ values were 9.63 *µ*g cm^−2^ and 1096 mg litre^−1^, respectively, against *Hyalomma dromedarii *larvae	Saudi Arabia	[143]
		Latex	Found to be acaricidal	Egypt	[148]

*Calpurnia aurea*	Fabaceae/Papilionaceae/Leguminosae (Hardy annual legume/pea family)	Leaf and bark	Juice mixed with spice of *Capsicum* spp.	Ethiopia	[149]

*Cinnamomum camphora* (camphor plant)	Lauraceae The Laurel family		Acaricide, essential oils repellent against *Ixodes* spp.	USA	[150]

*Cannabis sativa *L.(ganja/marijuana, marihuana, hemp, hashish, pot)	Cannabaceae (hemp family)	Fresh leaf	Topical application of crude ethanol extracts. Being toxic and inhabitant of oviposition and embryogenesis of *Boophilus microplus* Canst.	Jamaica	[58]

*Capsicum annum *L.(scotch bonnet/pimento or sweet pepper)	Solanaceae (lemon pepper/nightshade/potato family)	Fresh leaf	Topical application of crude ethanol extracts. Being Toxic and inhabitant of oviposition and embryogenesis of *Boophilus microplus* Canst.	Jamaica	[58]

*Capsicum *spp.	Solanaceae (lemon pepper/nightshade/potato family)	Fruits/leaves	Spice mixed with juice of leaf and bark from *Calpurnia aurea* to form an acaricide	Ethiopia	[149]

*Calpurnia aurea* L.	Fabaceae/Papilionaceae/Leguminosae/ Papilionaceae (hardy annual, legume/pea/bean family)	Leaf and bark	Spice mixed with juice of leaf and bark from *Calpurnia aurea* to form an acaricide	Ethiopia	[149]

*Carduus leptacanthus *Fresen.	Asteraceae (also known as Compositae or daisy family)		Acaricide	Rwanda	[151]

*Cassia tora* L.	Caesalpiniaceae/Leguminosae/ Papilionaceae/Fabaceae (hardy annual, legume bean/pea family)	Leaf	Juice of smashed leaves orally	India	[152]

*Cassia occidentalis* L./*Senna occidentalis* L. (coffee senna/coffeeweed)	Caesalpiniaceae/Leguminosae/ Papilionaceae/Fabaceae, (Hardy annual – legume Bean/pea Family)	Fresh leaf	Topical application of crude ethanol extracts. Being toxic and inhabitant of oviposition of *Boophilus microplus* Canst.	Jamaica	[58]

*Catharanthus roseus* L. (Madagascar periwinkle)	Apocynaceae (dogbane family)	Fresh leaf	Topical application of crude ethanol extracts. Being toxic and inhabitant of oviposition and embryogenesis of *Boophilus microplus* Canst.	Jamaica	[58]

Cecropia peltata L. (trumpet tree)	Cecropiaceae (previously included in the family Moraceae, mulberry family)	Fresh leaf	Topical application of crude ethanol extracts. Being toxic and inhabitant of oviposition and embryogenesis of *Boophilus microplus* Canst.	Jamaica	[58]

*Cenchrus ciliaris *L. (buffel grass)	Poaceae (the grass family)	Whole plant	Repellent	South America	[113]

*Chamaecyparis lawsoniana* (A. Murr.) Parl. (Port Orford cedar)	Cupressaceae (cypress family)	Stump oil	Toxic to nymphal and larval ticks (*Ixodes scapularis* (Say)) (LC50 = 0.487 and 0.041% wt : vol, resp.)	USA	[139]

*Chamaecyparis nootkatensis* (D. Don) Spach (Alaska yellow-cedar)	Cupressaceae (cypress family)	Heartwood and leaves	Toxic to nymphal and larval ticks (*Ixodes scapularis* (Say)) (LC50 = 0.151 and 0.007% wt : vol, resp.)	USA	[139]

*Chebliswo*-plant in Pokot vernacular in Kenya		Root and Leaf	Solution of smashed parts	Kenya	[153]

*Chenopodium ambrosioides *L. Mexican tea	Chenopodiaceae (goose-foot family)	—	—	Rwanda	[151]

*Chenopodium ugandae*	Chenopodiaceae (goose-foot family)	—	—	Uganda	[151]

*Chrysanthemum cinerariaefolium* L.	Asteraceae (also known as Compositae or daisy family)	Flowers	Pyrethrins act as acaricide/toxicant/repellent	USA	[150]

Citronella plants	Myrtaceae (Poaceae or Gramineae)	Leaf	Extracts-tick repellents against *Ixodes *spp.	USA	[154]

*Citrullus lanatus* Thunb.	Cucurbitaceae (cucumber family)	Fruit		Zimbabwe	[18, 105]

Citrus aurantium L.(Seville orange)	Rutaceae (rue family)	Fresh leaf	Topical application of crude ethanol extracts. Being toxic and inhabitant of oviposition and embryogenesis of *Boophilus microplus* Canst.	Jamaica	[58]

*Cleome hirta* (Klotzsch.) Oliv.	Cleomaceae	Aerial parts/oil	Repellent/toxic/killer of ticks *(Rhipicephalus appendiculatus)*	Kenya	[155]

*Cleome gynandra* (Cleome)	Cleomaceae	Leaves	Repellents and acaricides for certain larval, nymphal, and adult ticks	South Africa	http://www.daff.gov.za/docs/brochures/cleome.pdf

*Clerodendrum glabrum* E. Mey.	Lamiaceae	Leaf	Acetone extracts showed relatively high repellency activity against *Rhipicephalus appendiculatus* ticks	South Africa	[128]

*Commiphora swynnertonii *Burtt.	Burseraceae (copal family and/or torchwood family)	Gum resin	Repellency of their essential oil	Kenya	[104]

*Commiphora erythraea *Engler.	Burseraceae (copal family and/or torchwood family)	Gum resin/viscous oil/pure components/hexane extract	Smear paste of camel urine and gum resin/toxic/repellent/larvicide	Kenya	[67, 116, 156, 157]

Commiphora *holtziana*	Burseraceae (copal family and/or torchwood family)	Gum, bark, and leaf	Repellent	Kenya	[117]

*Commiphora incisa*	Burseraceae (copal family and/or torchwood family)	Gum resin	Smear paste of camel urine and gum resin	Kenya	[67]

*Commiphora merkeri* Engl.	Burseraceae (copal family and/or torchwood family)	Gum, bark, and leaf	Acaricide		[158]

*Commiphora myrrh* Jacq. (*Commiphora abyssinica* (Nees) Engl., *Commiphora myrrha* (Nees) Engl., and *Commiphora schimperi* (Nees) Engl.) (all these plants are sources of Myrrh)	Burseraceae (copal family and/or torchwood family)	Gum resin/oil/pure components	Toxic/Repellent	Kenya	[116, 156]
		Oleoresin gum (crude myrrh)	Myrrh essential oil/oil of Heerabol Myrrh (bola, myrrha and gum, common, and hirabol myrrh), repellent against *Ixodes *spp.	Mecklenburg County, North Carolina, USA	[125]

*Commiphora molmol* Engler.	Burseraceae (copal family and/or torchwood family)	Myrrh	LC_50_ of Myrrh extract caused death of fowl tick *Argas persicus* by destroying the epithelial gut of cells	Egypt	[159]

*Commiphora tenuis*	Burseraceae (copal family)	Larger leaf	Toxic/repellent when rubbed on camels' coats	Kenya	Wanzala-Personal experience with Somali pastoralists

*Crotalaria retusa* L. (rattle weed)	Caesalpiniaceae/Leguminosae/Papilionaceae/ Fabaceae, (Hardy annual, legume bean/pea family)	Fresh leaf	Topical application of crude ethanol extracts. Being toxic and inhabitant of oviposition and embryogenesis of *Boophilus microplus* Canst.	Jamaica	[58]

*Cuscuta americana *L. (love bush/weed)	Cuscutaceae (Convolvulaceae) (dodder family)	Fresh leaf	Topical application of crude ethanol extracts. Being toxic and inhabitant of oviposition and embryogenesis of *Boophilus microplus* Canst.	Jamaica	[58]

*Cycloptis semicordata* L. (tall fern)	Polypodiaceae (polypody/fern family)	Fresh leaf	Topical application of crude ethanol extracts. Being toxic and inhabitant of oviposition and embryogenesis of *Boophilus microplus* Canst.	Jamaica	[58]

*Cymbopogon flexuosus* (Nees ex Stend.) Wats. (lemon grass)	Gramineae (Poaceae) (grass family)	Leaf	A blend with marjoram grass and tea tree essential oils forms antitick repellent spray	New Zealand	[160]

*Cymbopogon martinii* stapf var. *motia*/(Roxb.) Wats. var. *motia* Burk.	Gramineae (Poaceae) (grass family)	Leaf/flower	Palmarosa essential oil (Turkish *Geranium*/*Andropogon*/Nepal), tick repellent against *Ixodes *spp.	Mecklenburg County, North Carolina, USA	[125]

*Cymbopogon nardus *R. *(Andropogon nardus)* (citronella grass, lemon grass, *nardus*)	Gramineae (Poaceae) (grass family)	Leaf/flower	Citronella essential oil (lemon balm, Sri Lanka or Lenabatu citronella), tick repellent against *Ixodes *spp.	Mecklenburg County, North Carolina, USA	[125]

*Cynodon dactylon *(star grass)	Poaceae (grass family)	Whole plant	Weak toxic/repellent	South America	[108]

*Datura stramonium* L.	Solanaceae (nightshade family)	Seed/fruit	Acaricide	Rwanda	[151]

*Delphinium brunonianum* Royle	Ranunculaceae (Helleboraceae) (buttercup family)	Leaf	Juices of leaves used to destroy ticks	USA	[18, 161]

*Derris elliptica* (Sweet) Benth.	Fabaceae/Papilionaceae/Leguminosae (hardy annual, legume/pea family)	Root powder	Aqueous solution mixed with soft soap to make an acaricide	USA	[18, 150, 162–164]

*Digitalis purpurea* L. (purple foxglove)	Scrophulariaceae (Figwort family)	A cardiac glycosidal (digitoxin) extract	Contact and dipping LC_50_ values were 4.08 *µ*g cm^−2^ and 409.9 mg litre^−1^, respectively, against *Hyalomma dromedarii *larvae	Saudi Arabia	[143]

*Dioscorea polygonoides* Willott (wild yam)	Dioscoreaceae (yam family)	Fresh leaf	Topical application of crude ethanol extracts. Being toxic and inhabitant of oviposition and embryogenesis of *Boophilus microplus* Canst.	Jamaica	[58]

*Diplophyllum africanum* Turcz.	Scapaniaceae (Diplophyllaceae)	Whole plant	Acaricide	Rwanda	[151]

*Dioscorea dumetorum* (Kunth) Pax.	Dioscoreaceae (yam family)	Root	Roots crushed in water to form a solution	Tanzania	[165, 166]

*Dissotis throthae*	Melastomataceae (melastome family)		—	Rwanda	[151]

*Ervatamia divaricate* L. (Burkill.) (coffee rose)	Apocynaceae	Fresh leaf	Topical application of crude ethanol extracts. Being toxic and inhabitant of oviposition and embryogenesis of *Boophilus microplus* Canst.	Jamaica	[58]

*Erythrina corallodendron *L. (Spanish maschette)	Caesalpiniaceae/Leguminosae/Papilionaceae/ Fabaceae (hardy annual, legume bean/pea family)	Fresh leaf	Topical application of crude ethanol extracts. Being Toxic and inhabitant of oviposition and embryogenesis of *Boophilus microplus* Canst.	Jamaica	[58]

*Eucalyptus* spp. (gum trees)	Myrtaceae (myrtle family)	Leaf and branch	Eucalyptus essential oil mixed with *S. Nigra* leaf extract make a repellent against *Ixodes* spp.	USA	[18, 167]

*Eucalyptus* spp. (gum trees)	Myrtaceae (myrtle family)	Leaf and branch	Plant oil as an acaricide	USA	[167]

*Eucalyptus* spp. (gum trees)	Myrtaceae (myrtle family)	Leaf and branch	Eucalyptus essential oil toxic to nymphal and larval ticks (*Ixodes scapularis* (Say)) at >2% concentration (wt : vol)	USA	[139]

*Eucalyptus globulus* Labill. (Tasmanian bluegum, eucalypt, and fever tree)	Myrtaceae (myrtle family)	Leaf and branch	Eucalyptus essential oil, Tick repellent against *Ixodes *spp.	Mecklenburg County, North Carolina, USA	[125]
		Leaf	Leaf decoction is traditionally used for repelling insects and vermin	USA	[168]

*Eupatorium odoratum* Penny Clifford (*Chromolaena odorata* (L.) RM King and H. Robinson) (Jack in the bush)	Asteraceae (daisy/aster family)	Fresh leaf	Topical application of crude ethanol extracts. Being toxic and inhabitant of oviposition and embryogenesis of *Boophilus microplus* Canst.	Jamaica	[58]

*Eupatorium villosum *(L.) George R. (bitter bush)	Asteraceae (daisy/aster family)	Fresh leaf	Topical application of crude ethanol extracts. Being toxic and inhabitant of embryogenesis of *Boophilus microplus* Canst.	Jamaica	[58]

*Euphorbia aegyptiaca* Boiss.	Euphorbiaceae (spurge family)	Aerial parts	Diethyl ether, ethyl acetate, hexane, and ethanol extracts showed toxicity against larvae of *Hyalomma dromedarii* Koch, 1844	Egypt	[138]

*Euphorbia candelabrum*	Euphorbiaceae (spurge family)	Latex	Latex as toxic/killer/acaricide	Kenya	[140]

*Euphorbia obovalifolia*	Euphorbiaceae (spurge family)	Latex	Acaricide	Ethiopia	[149]

*Fagara microcarpa* (Griseb.) Krug & Urb. (bitter bush)	Rutaceae (citrus family)	Fresh leaf	Topical application of crude ethanol extracts. Being toxic and inhabitant of oviposition and embryogenesis of *Boophilus microplus* Canst.	Jamaica	[58]

*Ficus brachypoda*	Moraceae	Latex	Acaricide	Ethiopia	[149]

*Ficus* cfr. *burkei*	Moraceae		Acaricide	Rwanda	[151]

*Foeniculum vulgare *P. Mill. (Florence fennel, finocchio, anise, and sweet fennel)	Umbelliferae/Apiaceae (carrot family)	Root	Toxic to nymphal ticks (*Ixodes scapularis* (Say)) (LC50 = 0.744% wt : vol)	USA	[139]

*Francoeuria crispa* (Forsk.) Cass. (Francoeuria)	Asteraceae (daisy/aster family)	Aerial parts	Diethyl ether, ethyl acetate, hexane, and ethanol extracts showed toxicity against larvae of *Hyalomma dromedarii* Koch, 1844	Egypt	[138]

*Gliricidia sepium* Jacq. (Aaron's rod)	Caesalpiniaceae/Leguminosae/Papilionaceae/ Fabaceae (hardy annual, legume bean/pea family)	Fresh leaf	Topical application of crude ethanol extracts. Being toxic and inhabitant of oviposition and embryogenesis of *Boophilus microplus* Canst.	Jamaica	[58]

*Gnidia kraussiana *Meissner	Thymelaeaceae	Root	Acaricide	Rwanda	[151]

*Gynandropsis gynandra *(L.) Briq. (Chisaka-Luhya, Ejobyo-Luganda, and Akeyo-Luo)	Capparidaceae	Aerial parts/essential oil	Repellent/toxic/killer	Kenya	[44, 104, 109, 118, 120]

*Haplophyllum tuberculatum* (Forsskål) A. H. L. Jussieu	Rutaceae	Aerial parts	Essential oils showed toxicity effects to the larvae of *Hyalomma dromedarii* Koch, 1844 and *Argas persicus* Oken, 1818, adults	Egypt	[138]

*Haematoxylum campechianum *L. (logwood)	Caesalpiniaceae/Leguminosae/Papilionaceae/ Fabaceae (hardy annual, legume bean/pea family)	Fresh leaf	Topical application of crude ethanol extracts. Being toxic and inhabitant of oviposition of *Boophilus microplus* Canst.	Jamaica	[58]

*Hedeoma pulegioides*L. Pers. (American Pennyroyal, mock pennyroyal, squaw mint, tickweed, stinking balm, mosquito plant)	Lamiaceae previously known as Labiatae (dead-nettle or mint family)	Leaf/flower	Essential oil of American pennyroyal, tick repellent against *Ixodes* spp.	USA	[125, 169, 170]

*Hibiscus rosa-sinensis *L. (shoe black)	Malvaceae (mallow family)	Leaf	Topical application of crude ethanol extracts. Being toxic and inhabitant of oviposition and embryogenesis of *Boophilus microplus* Canst.	Jamaica	[58]

*Hyacinthoides hispanica* (P. Mill.) Rothm. (*Endymion hispanicus* (P. Mill.) Chouard) (bluebells, Spanish bluebells, Spanish squill)	Liliaceae (lily family)	Whole plant	It is antitick plant and confidently help heal the problems brought about by ticks	USA and South Africa	Wanzala's personal communication with Annie Berthold-Bond in USA

*Hydnora johannis* Beccari	Hydnoraceae	Whole plant	Acaricide	Rwanda	[151]

*Hyparrhenia rufa *(jaragua grass)	Poaceae (grass family)	Whole plant	Weak toxic/repellent	South America	[128]

*Hyptis verticillata*	Lamiaceae previously known as Labiatae (dead-nettle or mint family)		Chemosterilant that inhibits oviposition and egg hatching	Central America	[171]

*Impatiens stuhlmannii *Warb.	Balsaminaceae (balsam, impatiens family)	Leaf	Acaricide	Rwanda	[151]

*Iphiona rotundifolia*	Asteraceae (also known as Compositae or daisy family)	Leaf	An acaricide infusion made from leaf	Somali	[61]

*Jatropha curcas* L. (Barbados nut, purging nut, and physic nut)	Euphorbiaceae (spurge family)	Leaf	Infusion had a dipping and topical toxicity effect against *Rhipicephalus appendiculatus* ticks. Methanol extract repelled the ticks also	South Africa	[128]

*Juglans nigra* L.	Juglandaceae	Whole plant	Repellent against *Ixodes* spp.	USA	[18, 167]

*Juniperus occidentalis *L (Western juniper)	Cupressaceae (cypress family)	Heartwood and leaves	Toxic to nymphal and larval ticks (*Ixodes scapularis* (Say)) (LC50 = 0.633 and 0.073% wt : vol, resp.)	USA	[139]

*Juniperus virginiana* L (Eastern red cedar)	Cupressaceae (cypress family)	Heartwood and leaves	Toxic to nymphal and larval ticks (*Ixodes scapularis* (Say)) (LC50 = 0.328 and 0.001% wt : vol, resp.)	USA	[139]

*Justicia pectoralis *L. (Fresh cut)	Acanthaceae (carpenter grass)	Fresh leaf	Topical application of crude ethanol extracts. Being toxic and inhabitant of embryogenesis of *Boophilus microplus* Canst.	Jamaica	[58]

*Kaliya,* Pokot vernacular		Fruit	Fruit juice	Kenya	[153]

*Lantana involucrata *Roxb. (wild mint)	Verbenaceae (vervain family)	Fresh leaf	Topical application of crude ethanol extracts. Being toxic and inhabitant of oviposition and embryogenesis of *Boophilus microplus* Canst.	Jamaica	[58]

*Laurencia obtusa* (Hudson) J. V. Lamouroux, 1813	Rhodomelaceae	Fresh leaf	Topical application of crude ethanol extract affected the survival of engorged and adult female *Boophilus microplus* Canst. and inhibited its oviposition and embryogenesis	Jamaica	[172]

*Lavandula angustifolia (L. officinalis, L. spica, *and* L. vera)*	Lamiaceae previously known as Labiatae (dead-nettle or mint family)	Leaf/flower	Lavender essential oil, tick repellent against *Ixodes *spp.	Mecklenburg County, North Carolina, USA	[125]

*Lepidium sativum*	Brassicaceae (cabbage family)	Seed	Crushed seeds mixed with cattle faeces and smeared on cattle	Ethiopia	[149]

Liagora* elongate* *Liagora farinosa* J. V. Lamouroux 1816	Liagoraceae (rhodophytes)	Whole plant	Topical application of crude ethanol extract affected the survival of engorged and adult female *Boophilus microplus* Canst. and inhibited its oviposition and embryogenesis	Jamaica	[172]

*Lippia alba* L. (colic Mint)	Verbenaceae (vervain family)	Fresh leaf	Topical application of crude ethanol extracts. Being toxic and inhabitant of oviposition and embryogenesis of *Boophilus microplus* Canst.	Jamaica	[58]

*Lonchocarpus laxiflorus*	Fabaceae/Papilionaceae/Leguminosae (hardy annual, legume/pea family)	Floral parts	Rotenoids act as acaricide	USA	[150]

*Lupinus mutabilis* Sweet (tarwi lupine/Adeans lupines/tarwi plant)	Fabaceae/Papilionaceae/Leguminosae (hardy annual, legume/pea family)	—	Acaricide	Europe	[91]

*Majorana hortensis* Mönch (sweet marjoram)	Lamiaceae previously known as Labiatae (dead-nettle or mint family)	Leaf	A blend with lemon grass and tea tree essential oils forms antitick repellent spray	New Zealand	[160]

*Mammea americana* L.	Clusiaceae/Guttiferae	Fruit and leaf/seeds	Toxic, sap/infusion in water/powdered seeds/decoction of seeds	USA	[18, 173]

*Margaritaria discoidea* (Baill.) G. L. Webster (pheasant-berry, egossa red pear, or bushveld peacock-berry)	Phyllanthaceae (leaf-flower family)	Latex	Toxic/killer/acaricide. Oil hexane and water soluble extracts against the ticks *Rhipicephalus appendiculatus* and *Amblyomma variegatum*	Kenya	[174]

*Melaleuca alternifolia* Cheel. (tea tree oil)	Myrtaceae (myrtle family)	Leaf	A blend with lemon grass and marjoram essential oils forms antitick repellent spray	New Zealand	[160]

*Melia azedarach *L. (Chinaberry, Persian lilac tree)	Meliaceae (mahogany family)	Fruit	Extracts caused mortality of *Boophilus microplus* larvae and inhibited partially or totally egg production and embryogenesis in engorged females	South America, Brazil	[175]

*Melicoccus bijugatus *Jacq. Guinep. Synonym: *Melicocca bijuga *L.	Sapindaceae	Fresh leaf	Topical application of crude ethanol extracts. Being toxic and inhabitant of oviposition and embryogenesis of *Boophilus microplus* Canst.	Jamaica	[58]

*Melinis minutiflora *Beauv. (molasses grass)	Poaceae or Gramineae (the grass family)	Whole plant (grass)	Toxic/repellent. The plant repels ticks (*Rhipicephalus appendiculatus* and *Boophilus microplus*)	South America, Caribbean, Kenya, Brazil, Mexico, Colombia, Central Africa, Southern Africa	[107, 108, 119, 127, 130, 131, 134, 176], Cornell University Medicinal plants Homepage-2003

*Melissa officinalis* L. (lemon balm, balm, common balm, cytria, hashishat al nahil, kovanutu, ogulotu, seiyo-yama-hakka, sweet balm, toronjil, tronjan)	Lamiaceae previously known as Labiatae (dead-nettle or mint family)	Aerial parts	European pennyroyal essential oil, tick repellent against *Ixodes *spp.	Europe and USA, Mecklenburg County, North Carolina	[125]

*Mesembryanthemus forsskale* (Hochst)	Aizoaceae	Aerial parts	Diethyl ether, ethyl acetate, hexane, and ethanol extracts showed toxicity against larvae of *Hyalomma dromedarii* Koch, 1844	Egypt	[138]

*Mentha *×* piperita* L. (*M. balsamea* Willd.) (pepper mint)	Piperaceae		Oil repellents against *Ixodes* spp.	USA	[154]

*Mentha pulegium* L. (European pennyroyal, pulegium, run-by-the-ground, lurk-in-the-ditch, pudding grass, mosquito plant, fleabane, tickweed, squaw balm, squawmint tickweed)	Lamiaceae previously known as Labiatae (dead-nettle or mint family)	Aerial parts	European pennyroyal essential oil, tick repellent against *Ixodes *spp.	Europe and USA, Mecklenburg County, North Carolina	[125]

*Mimosa pudica *L. (shame mi lady or sensitive plant)	Mimosaceae/Fabaceae	Fresh leaf	Topical application of crude ethanol extracts. Being toxic and inhabitant of oviposition and embryogenesis of *Boophilus microplus* Canst.	Jamaica	[58]

*Momordica charantia* L. (wild cerasee)	Cucurbitaceae	Leaf	Topical application of crude ethanol extracts. Being toxic and inhabitant of oviposition and embryogenesis of *Boophilus microplus* Canst.	Jamaica	[58]

*Neorautanenia mitis* (A. Rich) Verdc.	Fabaceae/Papilionaceae/Leguminosae (hardy annual, legume/pea family)	Root	Acaricide	Rwanda	[151]

*Nerium oleander *L. (Oleander)	Apocynaceae	Fresh leaf	Topical application of crude ethanol extracts. Being toxic and inhabitant of oviposition and embryogenesis of *Boophilus microplus* Canst.	Jamaica	[58]

*Nicotiana tabacum* L., *N. rustica, and N. glutinosa*	Solanaceae (nightshade family)	Fresh leaf	Leaf extract applied as acaricide. Toxic and inhabitant of oviposition and embryogenesis of *Boophilus microplus* Canst.	USA, Jamaica, and Kenya	[18, 43, 58, 67, 150, 167]

*Nicotiana tabacum* L. (Tobacco)	Solanaceae (nightshade family)	Aerial part	Aqueous extracts had immediate effect on mortality of engorged *Rhipicephalus haemaphysaloides* ticks and its fecundity production of females	India	[177]
		Leaf	Add Magadi soda to the leaf to make *Kupetaba,* antifeedant/growth disrupting/toxic/antiovipositant	Kenya	[43]
		Whole plant	A concoction mixed with sodom apple *(Solanum incanum)* to make an effective acaricide against brown ear tick *(Rhipicephalus appendiculatus)*, red-legged tick *(Rhipicephalus evertsii evertsi)*, *Boophilus decoloratus,* and bont tick (*Amblyomma *species)	Kenya (Samburu pastoralists) in Baragoi	[129]

*Ocimum micranthum *Wild. (wild parsley)	Lamiaceae previously known as Labiatae (dead-nettle or mint family)	Leaf	Topical application of crude ethanol extracts. Being toxic and inhabitant of oviposition and embryogenesis of *Boophilus microplus* Canst.	Jamaica	[58]

*Ocimum suave *Willd.	Lamiaceae previously known as Labiatae (dead-nettle or mint family)	Leaf	Oil as repellent/acaricide	Kenya, Tanzania	[178–180]

*Olea europaea* subsp. *Cuspidata* (African olive tree)	Oleaceae (olive family)	Whole plant	A concoction mixed with Ilkerereai *(Cadia purpurea)* make effective acaricide against brown ear tick (*Rhipicephalus appendiculatus*), red-legged tick (*Rhipicephalus evertsii evertsi*), *Boophilus decoloratus*, and bont tick (*Amblyomma *species)	Kenya (Samburu pastoralists) in Baragoi	[129]

*Oreopanax capitatus *Jacq. (Aralia)	Araliaceae	Fresh leaf	Topical application of crude ethanol extracts. Being toxic and inhabitant of oviposition and embryogenesis of *Boophilus microplus* Canst.	Jamaica	[58]

*Padina vickerisiae*	Phaeophyceae (brown seaweeds)	Whole plant	Topical application of crude ethanol extract affected the survival of engorged and adult female *Boophilus microplus* Canst. and inhibited its oviposition and embryogenesis	Jamaica	[172]

*Peganum harmala* L.	Zygophyllaceae	Aerial parts	Extracts of petroleum ether, chloroform, ethyl acetate, and ethanol showed toxicity effects on engorged females of *Boophilus annulatus* Say, 1821	Egypt	[138]

*Pelargonium graveolens* L'Hérit. or *P. Odoratissimum *(lemon plant/rose geranium/sweet scented geranium)	Geraniaceae (the stork's bill family)	Leaf/flower	Rose geranium essential oil, tick repellent against *Ixodes* spp.	Mecklenburg County, North Carolina, USA	[125]

*Pennisetum clandestinum *(Kikuyu grass)	Gramineae/Poaceae (grass family)	Whole plant	Weak toxic/repellent	South America	[108]

*Pennisetum typhoides*	Gramineae/Poaceae (grass family)	Corn and stem	Powder/dust	Southern Africa and Niger	[127, 181]

*Petiveria alliacea* L. (Guinea hen)	Phytolaccaceae	Leaf, root	Topical application of crude ethanol extracts. Being toxic and inhabitant of oviposition and embryogenesis of *Boophilus microplus* Canst. Crude ethanol extracts exhibit some repellent activity against *B. Microplus* Canst. Dibenzyltrisulfide, a compound in the roots of *P. alliacea, *is acaricidal	Jamaica; Central and South America, Caribbean and Africa	[58, 182] Cornell University Medicinal plants Homepage-2003

*Peucedanum angolense *(Welw.)	Apiaceae (carrot family)	Leaf	Acaricide	Rwanda	[151]

*Physostigma mesoponticum* Taub.	Fabaceae/Papilionaceae/Leguminosae (hardy annual, legume/pea family)	Tuber, leaf, bark, root	An infusion	Malawi	[96]

*Phytolacca dodecandra *L'Herit.	Phytolaccaceae	Leaf	Juice; acaricide	Rwanda, Ethiopia	[149, 151]

*Pimenta dioica* L. (pimento)	Myrtaceae	Fresh leaf	Topical application of crude ethanol extracts. Being toxic and inhabitant of oviposition and embryogenesis of *Boophilus microplus* Canst.	Jamaica	[58]

*Pimenta racemosa* (West Indian bay tree, bay rum tree, wild cinnamon, and bayberry)	Myrtaceae	Leaf	Bay essential oil, repellent against ticks (*Ixodes* spp.)	Mecklenburg County, North Carolina, USA	[125]

*Piper amalago* L. (black jointer)	Piperaceae	Fresh leaf	Topical application of crude ethanol extracts. Being toxic and inhabitant of oviposition and embryogenesis of *Boophilus microplus* Canst.	Jamaica	[58]

*Piper auritum* H. B. & K.	Piperaceae	Whole plant parts	Juice	USA	[18]

*Piper auritum* H. B. & K.	Piperaceae	Whole plant	Juice	Central America	[183]

*Piper capense* L. f.	Piperaceae	Leaf	Acaricide	Rwanda	[151]

*Piqueria trinervia* Cav.	Compositae	Leaf/flower/root	Piquerols A and B as acaricide against *Boophilus microplus* Canst.	South America	[184]

*Pongamia pinnata *Vent. (Indian beech, Pongam oil tree)	Fabaceae/Papilionaceae/Leguminosae (hardy annual, legume/pea family)	Seed	Pongamia essential oil is used as acaricide against *Boophilus microplus* Canst.	India	[145]

*Pseudotsuga menziesii* (Mirbel) Franco var. *menziesii* (formerly *P. taxifolia)* (Douglas-fir, Douglasfir)	Pinaceae (pine family)	Wood pitch	Toxic to nymphal and larval ticks (*Ixodes scapularis* (Say)) at >2% concentration (wt : vol)	USA	[139]

*Psiadia punctulata* (DC) Vatke	Asteraceae/Compositae (daisy family)	Whole plant	A concoction mixed with aloe *(Aloe secundiflora)* effective acaricide against brown ear tick (*Rhipicephalus appendiculatus*), red-legged tick (*Rhipicephalus evertsii evertsi*), *Boophilus decoloratus*, and bont tick (*Amblyomma *species)	Kenya (Samburu pastoralists) in Baragoi	[129]

*Ptaeroxylon obliquum* Radlk	Ptaeroxylaceae		An infusion of the powder as a wash	Southern Africa	[18, 127]

*Ranunculus multifidus* Forsk.	Ranunculaceae (buttercup family)	Fruit	Acaricide	Rwanda	[151]

*Reaumuria hirtella* (Jaub. & Spach)	Tamaricaceae	Aerial parts	Diethyl ether, ethyl acetate, hexane, and ethanol extracts showed toxicity against larvae of *Hyalomma dromedarii* Koch, 1844	Egypt	[138]

*Rhoicissus tridentata*	Vitaceae	Plant parts	Acaricide	Rwanda	[151]

*Ricinus communis* L. (castor oil plant)	Euphorbiaceae (spurge family)	Leaf	Topical application of crude ethanol extracts. Being toxic and inhabitant of oviposition and embryogenesis of *Boophilus microplus* Canst.	Jamaica	[58]
		Seed	Custard seed oil as an acaricide	India	[144]
		Leaf	Dichloromethane extracts were repellent to *Rhipicephalus appendiculatus*	South Africa	[128]

*Rosmarinus officinalis* L. (rosemary)	Lamiaceae previously known as Labiatae (dead-nettle or mint family)	Leaf	Rosemary essential oil, repellent against ticks (*Ixodes* spp.)	Mecklenburg County, North Carolina, USA	[125]

*Stylosanthes scabra* cv. Fitzroy or Seca	Fabaceae (hardy annual, legume/pea family)	Whole plant (grass)	Toxic to *Boophilus microplus* Canst.	South America	[110]

*Salvia serotina* L./Wild. (little woman/chicken weed)	Lamiaceae previously known as Labiatae (dead-nettle or mint family)	Fresh leaf	Topical application of crude ethanol extracts. Being toxic and inhabitant of oviposition and embryogenesis of *Boophilus microplus* Canst.	Jamaica	[58]

*Sambucus nigra* spp. *Canadensis (S. canadensis) *L. (American Elder or elderberry/European elder)	Adoxaceae/Caprifoliaceae (honeysuckle family)	Leaf	Extracts as acaricides	USA	[167]

*Sambucus nigra* spp. *Canadensis (S. canadensis) *L. (American Elder or elderberry/European elder)	Adoxaceae/Caprifoliaceae (honeysuckle family)	Leaf	Leaf extract mixed with tobacco dust and Eucalyptus oil	USA	[18, 167]

*Sclerocarya caffra* Sond.	Anacardiaceae	Fruit	Acaricide	South Africa/East Africa/Madagascar	[18, 127]

*Securidaca longipedunculata *Fres.	Polygalaceae	Plant	Acaricide	Rwanda	[151]

*Sequoia sempervirens *L. (redwood, coast redwood, and California redwood)	Taxodiaceae (bald cypress family)/Cupressaceae (cypress family)	Heartwood and leaves	Toxic to nymphal and larval ticks (*Ixodes scapularis* (Say)) (LC50 = 0.1.673 and 0.079% wt : vol, resp.)	USA	[139]

*Sequoiadendron giganteum *(Lindl.) J. Buchholz (giant sequoia, big tree, giant redwood)	Taxodiaceae	Heartwood and leaves	Toxic to nymphal and larval ticks (*Ixodes scapularis* (Say)) at >2% concentration (wt : vol)	USA	[139]

*Senna italica* subsp. *arachoides* (Mill.) Goora wall. (Italian senna)	Fabaceae (hardy annual, legume/pea family)	Root	The acaricidal activity of the ethyl acetate root extract increased significantly with concentration when tested against *Hyalomma marginatum rufipes*	South Africa	[185]

*Sida acuta *Burm. (broom weed)	Malvaceae	Fresh leaf	Topical application of crude ethanol extracts. Being toxic and inhabitant of oviposition and embryogenesis of *Boophilus microplus* Canst.	Jamaica	[58]

*Silybum marianum* (L.) Gaertn. (Milk thistle)	Asteraceae	Aerial parts	Extracts of petroleum ether, chloroform, ethyl acetate, and ethanol showed toxicity effects on engorged females of *Boophilus annulatus* Say, 1821	Egypt	[138]

*Simarouba glauca *DC. (Bitter wood)	Simaroubaceae (quassia family)	Fresh leaf	Topical application of crude ethanol extracts. Being toxic and inhabitant of oviposition and embryogenesis of *Boophilus microplus* Canst.	Jamaica	[58]

*Simmondsia chinensis* (Link) C. K. Schneid. (jojoba, goat nut, deer nut, pignut, wild hazel, quinine nut, coffeeberry, or gray box bush)	Simmondsiaceae	Aerial parts	Extracts caused motalities and affected reproductive physiology of the adult female tick, *Boophilus annulatus*	Egypt	[186]

*Solanum dasyphyllum* Schum. et Thonn.	Solanaceae (nightshade family)	Fruit, leaf, stem	Acaricide	Rwanda	[151]

*Solanum incanum *(sodom apple)	Solanaceae (nightshade family)	Fruit	Juice	Ethiopia	[149]
		Whole plant	A concoction mixed with tobacco (*Nicotiana tabacum*) to make an effective acaricide against brown ear tick *(Rhipicephalus appendiculatus)*, red-legged tick *(Rhipicephalus evertsii evertsi)*, *Boophilus decoloratus*, and bont tick (*Amblyomma *species)	Kenya (Samburu pastoralists) in Baragoi	[129]

*Sorghum bicolor* (L.) Moench (sorghum)	Poaceae (grass family)	Whole plant	Extracts caused motalities and affected reproductive physiology of the adult female tick, *Boophilus annulatus*	Egypt	[186]
		Aerial part	Affects livestock ticks	Kenya	[47]

*Spigelia anthelmia *L. (worm grass)	Loganiaceae (*Logania* family)	Fresh leaf	Topical application of crude ethanol extracts. Being toxic and inhabitant of oviposition and embryogenesis of *Boophilus microplus* Canst.	Jamaica	[58]

*Stachytarpheta jamaicensis* Wild. (vervine)	Verbenaceae (verbena/vervain family)	Fresh leaf	Topical application of crude ethanol extracts. Being toxic and inhabitant of oviposition and embryogenesis of *Boophilus microplus* Canst.	Jamaica	[58]

*Stemona collinsae *Craib.	Stemonaceae (*Stemona* family)	Whole plant	50% concentration of extract caused 100 and 93.33% mortalities of engorged nymphs and adults of *Boophilus microplus* Canst., respectively	Thailand	[187]

*Stemona tuberosa* Lour.	Stemonaceae (*Stemona* family)	Whole plant	5% of chlorhydric acid extracts killed larvae of *Rhipicephalus sanguineus*, *Boophilus microplus* and *Haemaphysalis intermedia* ixodid ticks	Vietnam	[188]

*Strychnos madagascariensis *Poir. (black monkeys)	Loganiaceae	Leaf	Infusion had a strong dipping and topical toxicity effect against *Rhipicephalus appendiculatus* ticks. The extract showed repellence activity against *R. appendiculatus*	South Africa	[128]

*Stylosanthes hamata* cv. Verano.	Fabaceae (pea family)	Whole plant (grass)	Toxic/repellent against *Boophilus microplus* Canst.	South America,	[189–192]
				Australia	[110, 111]

*Stylosanthes humilis *H. B. K.	Fabaceae (pea family)	Whole plant (grass)	Toxic/repellent against *Boophilus microplus* Canst.	South America	[189–192]

*Stylosanthes scabra *Vogel. (shrubby stylo)	Fabaceae (pea family)	Whole plant	Extracts caused a high larval mortality of *Rhipicephalus sanguineus*, *Boophilus microplus*, and *Haemaphysalis intermedia *ixodid ticks	India	[193]

*Stypopodium lobalum* (C. Agardh) Kützing.	Dictyotaceae (thalloid brown alga)	Whole plant	Topical application of crude ethanol extract affected the survival of engorged and adult female *Boophilus microplus* Canst. and inhibited its oviposition and embryogenesis	Jamaica	[172]

*Symphytum officinale *L. (Comfrey)	Boraginaceae (borage family)	Fresh leaf	Topical application of crude ethanol extracts. Being toxic and inhabitant of oviposition and embryogenesis of *Boophilus microplus* Canst.	Jamaica, USA	[58]

*Tabernaemontana johnstonii*	Apocynaceae	—	Acaricide	Rwanda	[151]

*Tagetes minuta *L. (marigold)	Asteraceae (daisy family).	Aerial parts	Extracted essential oil had significant repellent effect against *Rhipicephalus appendiculatus* adult ticks	Kenya	[47, 194]
			Extracted essential oil had significant repellent effect against *Hyalomma rufipes* adult ticks	South Africa	[195]

*Tagetes patula* French (marigold, dwarf marigold, or dwarf French marigold)	Asteraceae (daisy family)	—	—	Rwanda	[151]

*Tamarindus indicus* L. (tamarind)	Caesalpiniacae (gulmohar family)	Mature fruit	Water and 10% ethanol crude extracts caused mortality of engorged female, *Boophilus microplus* Canst. Organic acids in tamarind fruits (oxalic, malic, succinic, citric and tartaric acids) also caused mortality of *B. microplus* Canst.	Thailand	[196]

*Tanacetum vulgare*	Asteraceae ([also known as Compositae] daisy family)	Whole plant	Essential oils are acaricidal	Europe, Eastern North America	http://www.florahealth.com/home_int.cfm

*Taxodium distichum *(L.) L. C. Rich. (bald cypress and swamp Cypress)	Taxodiaceae (bald cypress family)	Heartwood and leaves	Toxic to nymphal and larval ticks (*Ixodes scapularis* (Say)) at >2% concentration (wt : vol)	USA	[139]

*Tephrosia leiocarpa *A. Gray	Fabaceae/Papilionaceae/Leguminosae (hardy annual, legume/pea family)	Root	Acaricide	North America	[197]

*Tephrosia vogelii* Hook F.	Fabaceae/Papilionaceae/Leguminosae (hardy annual, legume/pea family)	Leaf, root, pod, seed, bark, whole plant	Rotenoids present in an infusion acts like modern dips. Toxic to 1-, 2-, and 3-host ticks	Cameroon, USA, Malawi, Tanzania	[18, 150, 198–202]

*Thuja plicata* Donn ex D. Don in Lambert 1824 (Western/giant red cedar, giant arborvitae, shinglewood, canoe cedar)	Cupressaceae (cypress family)	Heartwood and leaves	Toxic to nymphal and larval ticks (*Ixodes scapularis* (Say)) (LC50 = 0.821 and 0.022% wt : vol, resp.)	USA	[139]

*Thylachium africanum *Lour.	Capparidaceae	Aerial parts/oil	Repellency of their essential oil	Kenya	[104]

*Turnera ulmifolia *L. (ramgoat dashalong)	Turneraceae	Fresh leaf	Topical application of crude ethanol extracts. Being toxic and inhabitant of oviposition and embryogenesis of *Boophilus microplus* Canst.	Jamaica	[58]

Turpentine, name applied to numerous semifluid, yellow or brownish oleoresins obtained from various coniferous trees in Asia, Europe, and America Turpentine, *Syncarpia glomulifera*	Myrtaceae	—	Acaricide	USA	[150]

*Urena lobata* L. (bur mallow)	Malvaceae	Fresh leaf	Topical application of crude ethanol extracts. Being toxic and inhabitant of oviposition and embryogenesis of *Boophilus microplus* Canst.	Jamaica	[58]

*Vernonia amygdalina* L. (bitter-leaf tree)	Asteraceae (also known as Compositae or daisy family)	Leaf	Juice	Ethiopia	[149]

*Vitex agnus-castus* L. (chaste tree, chasteberry, Abraham's balm, or monk's pepper)	Lamiaceae	Aerial parts	Ethanol, propylene carbonate, *Vitex agnus-castus* concentrate, topical application, repelling horseflies, flies and mosquitoes	Switzerland	[203]

*Wedelia trilobata *L. (yellow marigold)	Asteraceae (also known as Compositae or daisy family)	Fresh leaf	Topical application of crude ethanol extracts. Being toxic and inhabitant of oviposition and embryogenesis of *Boophilus microplus* Canst.	Jamaica	[58]

*Zingiber officinale *Wild. (Ginger)	Zingiberaceae	Fresh leaf	Topical application of crude ethanol extracts. Being toxic and inhabitant of oviposition and embryogenesis of *Boophilus microplus* Canst.	Jamaica	[58]
